# Quality of Life in Patients With Parkinson’s Disease: A Cross-Sectional Study

**DOI:** 10.7759/cureus.33989

**Published:** 2023-01-20

**Authors:** Noora Al-Khammash, Nujud Al-Jabri, Amal Albishi, Afaf Al-Onazi, Sharifa Aseeri, Faisal Alotaibi, Yagob Almazroua, Monirah Albloushi

**Affiliations:** 1 College of Nursing, King Saud University, Riyadh, SAU; 2 Neurosurgery, King Faisal Specialist Hospital and Research Centre, Riyadh, SAU; 3 Epidemiology, Saudi Parkinson’s Disease Society, Riyadh, SAU

**Keywords:** management, covid 19, quality of life, parkinson's disease, domains of health

## Abstract

Patients with Parkinson’s disease (PD) suffer from a range of physical, psychological, and social problems. The disease affects the quality of life (QOL) of the affected person. Several factors contribute to QOL, and these factors should be examined to develop appropriate strategies. This study aimed to determine the factors related to QOL in patients with PD. A cross-sectional, descriptive study was conducted using a tool with strong validity and reliability (39-Item Parkinson’s Disease Questionnaire (PDQ-39)) to assess the quality of life. Descriptive statistics were used to analyze the data, and non-parametric chi-square tests were applied to evaluate the relationship between QOL and the variables. Frequent hospital admissions, level of education, and marital status were among the factors that affected QOL. The ability to perform Ramadan fasting correlated with the degree of QOL. The coronavirus disease 2019 (COVID-19) pandemic has adversely affected the quality of life due to changes in access to medical care and medications. To improve QOL in patients with PD, a comprehensive approach is required in many healthcare domains that includes physiotherapy together with the conventional pharmacotherapy, other treatments, and psychological support.

## Introduction

Parkinson’s disease (PD) is a chronic disease of the central nervous system that is characterized by both physical and psychological symptoms [1]. Patients with PD suffer from depression, anxiety, apathy, and excessive daytime sleepiness, which affect their health-related quality of life (QOL). There is a need to identify and treat these symptoms to improve these patients’ health-related QOL [2].

Patients affected by PD have motor and non-motor symptoms, and treating these symptoms may improve their QOL. Receiving care from a multidisciplinary team also helps to improve QOL [3,4]. Patients with PD experience neuropsychiatric symptoms and mild cognitive impairment, which affect their QOL [5,6]. Progression of the disease may affect the psychosocial domain and effective coping skills, requiring a psychosocial adjustment for patients and caregivers. The degree of adjustment depends on the QOL, and this can benefit the caregivers of patients with PD [7].

QOL refers to the standard of health, comfort, and happiness experienced by an individual or group [8]. Living with PD is challenging as it impacts all aspects of a patient’s life. Starting with mild symptoms, the disease gradually progresses to a level where activities of daily living become difficult and affect the QOL. The decline in QOL can lead to stigma, worsening of cognition, and greater impairment in mobility, including activities of daily living [9]. Several factors influence the QOL in patients with parkinsonism. A study reported a decrease in the QOL of patients with PD, which was observed in both physical and psychological parameters [10,11].

There are different treatment modalities available to manage PD-related symptoms that result in improvement of the QOL in this patient group [12,13]. Failure to address QOL issues leads to negative consequences for the individual and their family. Therefore, it is important to address the factors influencing QOL and to take initiative to improve the patient’s QOL.

There are certain cultural differences around the world that may contribute to the assessment of QOL. Ramadan fasting is practiced in many countries around the world, and few studies have investigated the recommendations and the ability of PD patients to perform fasting [14,15]. No study has correlated Ramadan fasting to QOL. In addition, the COVID-19 pandemic has impacted many patients with PD in different aspects [16,17]. This study aimed first to assess the level of QOL in patients with PD, second to evaluate the relationship between the factors (i.e., determinants of fasting for Ramadan and COVID-19) influencing QOL in patients with PD, and lastly to determine the association between sociodemographic factors and QOL in patients with PD.

## Materials and methods

Study design and setting

This study has a cross-sectional, descriptive design. This design was selected to examine factors that influence QOL in patients with PD. The data were collected from multiple healthcare institutions in Saudi Arabia and from the Saudi Society for Parkinson’s Patients. Ethical approval and permission from the study sites was obtained before the data collection. These settings were chosen based on the availability of the samples for the study. The population of this study included patients diagnosed with PD. The participants were chosen based on their availability during the data collection period, and the non-probability convenience sampling method was used to select the participants.

Inclusion and exclusion criteria

Patients who were diagnosed with PD and were receiving treatment for PD were eligible to participate in the study. Patients with PD with severe comorbidities, such as stroke, bedridden, or other severe medical illnesses, contraindicate the questionnaire application. In addition, severe cognitive and patients with dementia were excluded from participating in the study.

Data source and tools

Data were collected using web-based questionnaires. The link was distributed to all patients who met the inclusion criteria and decided to participate in the study. Data were collected between April 2021 and May 2022. The consent form included detailed information about the purpose of the study, risks and benefits, and confidentiality of the participants’ information. Participants were only able to access the questionnaires after reading and signing the consent form. Participants were informed that their information would only be accessible to the research team. They were informed that their responses would be reported in aggregate form and would be destroyed following the publication of the study.

The data collection instrument consisted of four sections: demographic data, determinants of fasting for Ramadan, determinants of COVID-19, and a scale to assess the QOL (PDQ-39). The demographic data form consisted of the basic demographic data about the participant, including age, gender, level of education, marital status, employment status, number of children, and initial diagnosis with PD. Two scales were developed for this study: determinants of fasting for Ramadan and determinants of COVID-19. Development of these scales required several steps: reviewing the literature, drafting the items, and sending the questionnaires to four subject matter experts in the fields of epidemiology, neuroscience, surgery, and nursing. Pilot testing was used to assess the clarity of the questionnaires. All the questionnaires were sent to 10 participants. After completing the data collection, Cronbach’s alpha of the determinants of fasting for Ramadan was .77, and Cronbach’s alpha of the determinants of COVID-19 was .70, indicating the reliability of these tools. The validity of the tools was not evaluated in this study.

The Parkinson's impact scale contains 10 items: Self (Positive), Self (Negative), Family Relationships, Community Relationships, Work, Travel, Leisure, Safety, Financial Security, and Sexuality [18,19]. The participants assessed the symptoms by self. Furthermore, the participants were allowed to complete the survey in their best state and in their worst state.

The study used the PDQ-39 questionnaire [20]. This standard tool is available online for research purposes. The tool consists of 39 items, rated on a five-point scale: never, occasionally, sometimes, often, always. The scale consists of eight dimensions: mobility, activities of daily living, emotional wellbeing, stigma, social support, cognitive impairment, communication, and bodily discomfort. The scores were transformed to a scale from 0 to 100. Higher scores indicated a poorer QOL. Due to our use of forced completion across all the items, missing data were 0% across the subscales. Cronbach’s alpha was used to measure the internal consistency reliability; a value of .89 was reported for the PDQ-39 after the translation. Cronbach’s alpha of the subscales ranged from .78 to .94.

Determinants of fasting for Ramadan

Participants were instructed to rate their degree of agreement regarding factors related to fasting for Ramadan after they had been diagnosed with PD. These factors were: difficulty with fasting, increase in Parkinson’s symptoms during Ramadan, routine changes in the dosing of medicines during Ramadan, changes to their treatment plan during Ramadan, and accessing emergency services while fasting for Ramadan. After grouping these items together, Cronbach’s alpha was .76.

Determinants of COVID-19

Participants provided their degree of agreement regarding the determinants of COVID-19. The items were related to the difficulty obtaining medical services and prescriptions during the pandemic, increases in Parkinson’s symptoms during the pandemic, differences in care, and fear of COVID-19. When these items were grouped together, Cronbach’s alpha was .71.

Statistical analysis

Data were entered and analyzed using the Statistical Package for Social Sciences version 28.0 (IBM Corp., Armonk, NY). Descriptive statistics (e.g., mean, standard deviation, percentage, and Cronbach’s alpha) and inferential statistics (Pearson’s product moment correlation and multiple linear regression) were used to determine the relationships between the variables of interest.

Ethical consideration

Institutional review board approval was obtained from King Saud University’s research ethics committee (KSU-HE-21-322). Participation in the study was voluntary. Participants were informed that they had the right to withdraw their participation at any time without any consequences. The contact information of the institutional review board and authors was provided to the participants.

## Results

The total sample size was 82. Most participants were aged between 51 and 64 years (40.2%) or older (34.1%). The majority of the participants (58.5%) were male, had a university degree (41.5%), and were married (79.3%), and 43.9% were diagnosed with PD over seven years prior to this study. In addition, more than one-half of the sample (56.1%) had other concomitant diseases. Table 1 presents additional demographic characteristics.

**Table 1:** Demographic and clinical characteristics of the study sample (N=82) n: number; %: percentage.

Factors		n		%
Age (years)	<35	9		11.0
	36–50	12		14.6
	51–64	33		40.2
	>65	28		34.1
Gender	Male	48		58.5
	Female	34		41.5
Education level	Illiterate	16	19.5	
	School	32	39	
	University	34	41.5	
Marital status	Single	9		11
	Married	65		79.3
	Widower	6		7.3
	Divorced	2		2.4
Number of children	1–4	28		34.1
	5–7	44		53.7
	>8	10		12.2
Employment status	Employed	69		84.1
	Unemployed	13		15.9
Initial diagnosis with Parkinson’s disease	<1 year	6		7.5
	1–3 years	17		20.7
	4–6 years	23		28
	>7 years	36		43.9
Other diseases	Yes	46		56.1
	No	36		43.9
Hospitalization due to Parkinson’s disease	Yes	27		32.9
	No	55		67.1

Quality of life in patients with Parkinson’s disease

The mean QOL score was 80.3 (SD = 37.9). The scores for the PDQ-39 dimensions are listed in Table 2. Regarding the dimensions of the PDQ-39, mobility was the top issue that threatened the QOL of patients with PD (M = 24.5, SD = 10.9), followed by activities of daily living (M = 13.3, SD = 7.6).

**Table 2:** Descriptive statistics for PDQ-39 and its subscales N: number; Std: Standard.

Descriptive Statistics						
	N	Minimum	Maximum	Mean	Std. Deviation	Cronbach’s Alpha
Quality of Life	82	14.00	150.00	80.3049	37.94137	.89
Mobility	82	1.00	40.00	24.5000	10.98512	.94
Activities of Daily Living	82	.00	24.00	13.3415	7.63232	.94
Emotional Wellbeing	82	.00	24.00	12.5732	7.18027	.93
Stigma	82	.00	16.00	5.9268	5.12505	.89
Social Support	82	.00	12.00	4.3659	3.78272	.84
Cognitions	82	.00	16.00	7.4390	4.57340	.89
Communication	82	.00	12.00	5.6707	3.82666	.87
Bodily Discomfort	82	1.00	12.00	6.4878	3.36006	.78

Determinants of fasting for Ramadan, COVID-19, and quality of life in patients with Parkinson’s disease

Determinants of fasting for Ramadan in patients with PD were significantly correlated with QOL (r = .366, p < .01). Participants had difficulty fasting during Ramadan, experienced an increase in Parkinson’s symptoms, changed the dosing of medicines or their treatment plan, and experienced decreased QOL (Table 3).

**Table 3:** Correlation between determinants of fasting for Ramadan, COVID-19, and quality of life N: Number; COVID-19: Coronavirus Disease 2019.

Predictors	Quality of Life		
	N	Pearson’s correlation	p-value
Determinants of Fasting for Ramadan	82	.366	< .01
Difficulty with fasting	82	.452	< .001
Increase in Parkinson’s symptoms	82	.414	< .001
Changing the doses of medicines	82	.245	.026
Changing in treatment plan	82	.238	.031
Accessing emergency services while fasting	82	.061	.591
Determinants of COVID-19	82	.581	< .01
Difficulty obtaining medical services	82	.566	< .001
Difficulty obtaining prescriptions	82	.366	< .001
Increase in Parkinson’s symptoms	82	.332	.002
Differences in receiving care during the pandemic	82	.371	< .001
Fear of contracting COVID-19	82	.374	< .001

Determinants of COVID-19 were significantly correlated with QOL in patients with PD (r = .58, p < .01). Participants had difficulty accessing medical services, experienced increased Parkinson’s symptoms, faced differences in care, were afraid of contracting COVID-19, and experienced decreased QOL. Additional information regarding the correlation between each item and QOL is presented in Table 3.

Relationship between participants’ characteristics and quality of life

Following regression analyses, the participants’ demographic and clinical characteristics explained 24.5% of the variance in QOL (R2 = .259, F (9, 72) = 2.72, p = .009) (Table 4). Among the variables entered in the model, level of education, marital status, and being hospitalized due to PD had a significant negative influence on patients’ QOL. Patients with a lower level of education had a lower QOL than their more educated counterparts ( = -.259, p < .001). Being single or divorced had a significant negative influence on QOL ( = -.482, p = .04). Hospitalization due to PD had a significant negative influence on QOL in patients with PD ( = -.742, p = .004).

**Table 4:** Multiple linear regression analysis of the participants’ characteristics and quality of life *p < .05; **p < .001 B^a^: coefficient; β^b^: Beta standardized coefficient; R^2^: coefficient of determination; F: f-test statistics; t: t-test.

Independent variables	Quality of Life		
	B^a^	β^b^	t
Age	-.021	-.021	-.136
Gender	-.155	-.080	-.665
Employment status	.389	.148	1.16
Level of education **	-.259	-.432	-3.50
Marital Status*	-.482	-.266	-2.04
Number of children	-.210	-.141	-1.05
Initial diagnosis with Parkinson’s disease	-.165	-.167	-1.36
Having other diseases	-.390	-.202	-1.69
Hospitalization due to Parkinson’s disease*	-.742	-.364	-3.00
Model summary	R^2^ = .259, F (9, 72) = 2.72, p = .009^*^		

## Discussion

PD is a complex neurological disorder due to its progressive nature, individual variations, and the variety of manifestations that arise during the disease. Despite the highly specialized information available in the scientific literature and the advancement of therapeutic options, there are several unmet patient needs [2,21]. The literature suggests that investigating symptoms and their impact on QOL in PD patients could help to improve the treatment options [22]. Understanding the disease burden in different geographical locations around the world will provide further understanding of the pathophysiology and impact of the disease. The prevalence of mild cognitive impairment in patients with PD ranges from 17% to 53% [5]. A regional study that aimed to increase the understanding of PD in different countries in the Middle East, North Africa, and South Asia (MENASA) region explored the impact of different factors on the QOL and healthcare needs of PD patients [23]. According to this study, environmental factors, unique genotype, and cultural influences may influence disease complications and QOL. Another study reported that anxiety and depression were the most overlooked non-motor symptoms in PD [6].

Motor and psychosocial dysfunction and psychiatric comorbidities (e.g., bradykinesia, rigidity, gait freezing, depression, fatigue, cognitive decline, and sleep disturbances) are common in PD patients, and these factors can decrease patients’ QOL. As expected, our study revealed that the QOL in general was affected by the disease, which is in line with other studies from different countries [24]. Education level was an important factor that adversely affected QOL in the present study. Similar results were reported in other studies where the lower education level of the participants correlated with their economic status [25,26]. Being unmarried had a negative impact on the QOL of participants with PD. In addition, repeated admissions to the hospital may indirectly indicate increased disease severity, and this factor was associated with decreased QOL scores in those patients.

The COVID-19 pandemic has impacted healthcare delivery, and this alone has adversely affected the care delivered to PD patients [17,27,28]. Isolation, altered medical supply, and poor access to healthcare adversely affect the QOL in PD patients. This effect was observed in our study in the following factors that negatively affected the QOL: limited access to medication; changes in care delivery methods, such as virtual clinics; and being afraid of contracting COVID-19. Furthermore, Ramadan fasting was determined to be an indirect indicator of QOL; increased difficulty in performing fasting was associated with decreased QOL. This could indirectly indicate advanced disease stage and severity or the nature of underlying motor and non-motor symptoms.

This is the first study to evaluate the QOL of patients with PD in Saudi Arabia. Although the study was based on a limited number of patients, the results provide insights into the comprehensive care requirements of patients with PD. To improve QOL, patients with PD require physical rehabilitation with conventional pharmacotherapy and other treatments, in addition to timely adjunctive psychotherapy. The limitations of the present study are that certain factors related to QOL, such as illness duration, disease severity, medical service system, and the treatment provided, were not analyzed. In addition, the study was limited by the use of the PDQ-39 without other instruments to measure more specific factors, the limited number of patients, and the nature of survey subjectivity.

## Conclusions

QOL in PD patients is determined by multiple domain-based factors. Frequent hospital admissions, level of education, and marital status are among the factors that affect QOL. The ability to perform Ramadan fasting correlated with the degree of QOL. The COVID-19 pandemic has adversely affected QOL due to changes in access to medical care and medications. To improve QOL in patients with PD, a comprehensive approach is required in many healthcare domains, including physiotherapy, conventional pharmacotherapy, other treatments, and psychological support.
